# The Black‐White Disparity in Preterm Birth: Race or Racism?

**DOI:** 10.1111/1468-0009.12625

**Published:** 2023-04-25

**Authors:** PAULA BRAVEMAN

**Affiliations:** ^1^ University of California San Francisco

**Keywords:** racism and health, preterm birth, racial disparities

## Abstract

**Policy Points**

- Racism is an upstream determinant of health that influences health through many midstream and downstream factors. This Perspective traces multiple plausible causal pathways from racism to preterm birth.
- Although the article focuses on the Black‐White disparity in preterm birth, a key population health indicator, it has implications for many other health outcomes.
- It is erroneous to assume by default that underlying biological differences explain racial disparities in health. Appropriate science‐based policies are needed to address racial disparities in health; this will require addressing racism.

Preterm birth (ptb)—birth before 37 completed weeks of pregnancy—is among the most important indicators of a population's health. It is the second most common cause of infant mortality in the US population overall^1^ and the leading cause among African Americans.^2^ PTB has serious adverse consequences not only in infancy but across the life course; it is a major cause of childhood developmental disability^3^ and a strong predictor of chronic disease in adulthood.^4^, ^5^ A large racial disparity in PTB has persisted for decades, with rates among African American/Black women approximately 1.5 to 1.6 times those among European American/White women.^6^

Unfortunately, the causes of both PTB and the racial disparity in PTB—which are not necessarily the same—are not well established. Despite lack of evidence and/or evidence to the contrary, widespread assumptions have been made about the racial disparity in PTB; as with many health outcomes, “race” has often been assumed by default and without evidence to reflect differences in underlying biology or behaviors.^7^, ^8^, ^9^ Evidence has been mounting over the past two decades that calls those assumptions into serious question, however, pointing to racism as a highly plausible upstream—i.e., fundamental—cause of the Black‐White disparity in PTB.^10^

This paper briefly reviews the evidence about biologically plausible causes of the racial disparity in PTB, focusing on racism and associated racial differences in experiences and exposures that are rarely measured in health studies. The terms “race” and “racial” are used here to refer to social groupings according to the large geographic area (often a continent) of an individual's ancestry. Geographic ancestry often correlates with observable superficial secondary physical characteristics such as skin color, facial features, or hair texture and with differences in the way that people are treated, but has not been associated with fundamental biological differences.^11^, ^12^, ^13^, ^14^, ^15^, ^16^

## What Is Known About the Causes of the Black‐White Disparity in PTB?

While much is unknown or uncertain, research has identified several explanations for the racial disparity in PTB that appear unlikely. Differences in the quality of prenatal care may play a role,^17^, ^18^, ^19^ although considerable research has indicated that the PTB disparity is not explained by differences in receipt of standard prenatal care;^20^, ^21^ use of tobacco, alcohol, or illicit drugs;^22^, ^23^, ^24^, ^25^, ^26^ or by current income or education.^27^ While socioeconomic measures (such as income and/or education) around the time of pregnancy consistently predict PTB rates among White women, this has not been the case among Black women. Furthermore, the racial disparity in PTB has been widest among women of high socioeconomic status and minimal or absent among economically disadvantaged women.^28^, ^29^ Infections have been strongly associated with PTB, and Black women have higher rates of prenatal infection;^30^, ^31^ treatment of the infections, however, has not consistently improved PTB rates.^30^, ^32^, ^33^, ^34^ This raises the question whether the infections, rather than being a cause, are a marker for something else, an underlying factor—such as stress‐induced inflammation and/or immunocompromise—that causes both infections and PTB.^33^, ^35^

Despite lack of supporting evidence, genetic differences have often been assumed to be the principal explanation for the racial disparity in PTB.^7^, ^8^, ^9^ This assumption has been reinforced in part because the disparity has generally persisted after adjustment for a mother's income and education around the time of pregnancy and in part because of failure to distinguish influences on PTB from influences on the racial disparity in PTB. A recent review of evidence by a panel of experts, including experts in genetics, concluded, however, that although genetic differences may contribute to PTB, they likely play a small, if any, role in the *racial disparity* in PTB.^10^, ^13^, ^36^, ^37^, ^38^ To account for the racial disparity in PTB, a cause—for example, a genetic variant—must not only influence PTB; it must also be more prevalent and/or have a greater effect size among one racial group. No such variant has been identified by large‐scale genomic studies.^10^ Gene‐environment interactions, however, may be important.^39^, ^40^, ^41^, ^42^ Potential mediators of epigenetic change include a range of social and environmental factors that are both associated with PTB risk and more prevalent among African American women, such as exposure to environmental toxins (due to environmental injustice), stress (e.g., due to experiences of racism and/or the economic hardship it produces), and diet (e.g., a recent study identified differences in gene expression associated not only with PTB but also with vitamin D insufficiency, which is more common among Black women).^43^

A large body of evidence indicates that social and environmental causes play an important role in both PTB^44^ and the racial disparity in PTB.^10^ Among the most compelling evidence of social rather than underlying biological causes is the observation of good birth outcomes among Black immigrants to the United States from Africa—that is, the absence of a racial disparity compared with their White counterparts.^38^, ^45^ If genetic differences were the cause of the disparity, one would expect to see worse, not better, birth outcomes among Black women who emigrate to the United States from Africa, whose genetic endowment would not be as “diluted” by admixture with European Americans as that of African Americans. The PTB disparity, furthermore, has often been found to vary with neighborhood social conditions.^28^, ^46^, ^47^, ^48^ Many studies have observed a consistent and important role for environmental toxins such as air or ground pollution,^49^, ^50^, ^51^, ^52^ which reflect the social policies driving environmental injustice. Some studies have observed no or a minimal racial disparity in PTB among poor women and the largest racial disparity among more socioeconomically advantaged women, suggesting the role of social factors.^28^, ^29^ Moreover, several studies have found associations between adverse birth outcomes and experiences of racial discrimination (e.g., unfair treatment, insults, threats, vigilance);^53^, ^54^, ^55^, ^56^ stress, discussed later in the article, is thought to mediate these associations. Nutrition also may contribute.^57^, ^58^ Many studies have associated stress and/or lack of social support (which can modify the effects of stress) with PTB.^27^, ^59^ The associations between stress and PTB, however, have not been consistent, which may reflect differences in the measurement of stress or the timing of measurement, for example, only during pregnancy versus throughout a woman's life before she became pregnant.^60^, ^61^ A number of researchers have called attention to the need to consider exposures and conditions experienced across a woman's entire life course, and particularly in early childhood, not only during pregnancy.^62^, ^63^, ^64^, ^65^, ^66^

## Racism Is a Biologically Plausible Upstream Cause of the PTB Disparity

Based on the totality of available evidence, many researchers have concluded that racism in many forms is a highly plausible upstream cause of the racial disparity in PTB.^7^, ^10^, ^27^, ^62^, ^67^, ^68^ Racism is more than just individual acts of race‐based discrimination. Camara Jones (2018) has defined *racism* as follows:

Racism is a system of structuring opportunity and assigning value based on the social interpretation of how one looks (which is what we call “race”), that unfairly disadvantages some individuals and communities, unfairly advantages other individuals and communities, and saps the strength of the whole society through the waste of human resources.^69^

**Figure 1:**
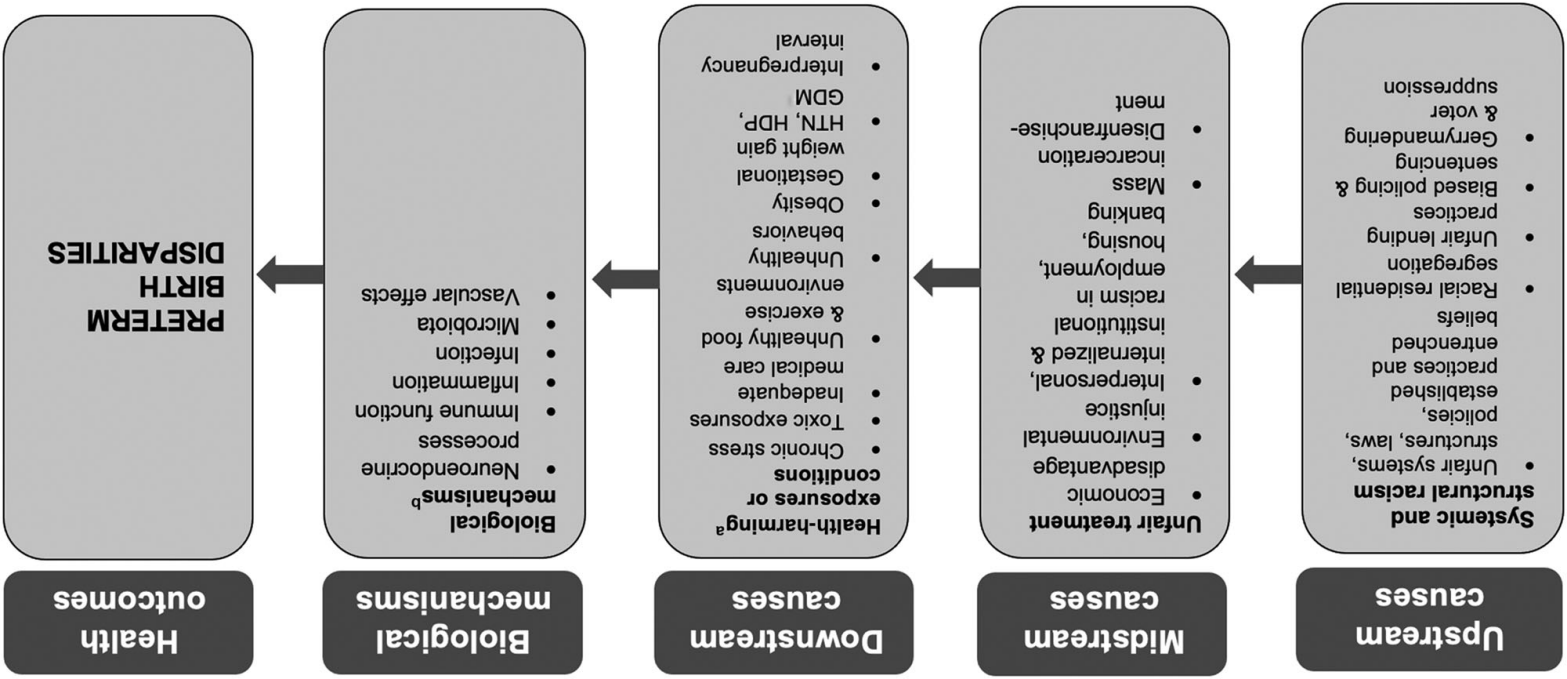
Racism Plausibly May Affect PTB As an Upstream Cause, Acting Through Midstream and Downstream Causes That More Directly Trigger the Physiologic Mechanisms Resulting in Racial Disparities in Preterm Birth **Abbreviations**: GDM, gestational diabetes mellitus; HDP, hypertensive disorders of pregnancy; HTN: hypertension. ^a^ Health‐harming (or lack of health‐promoting) exposures/conditions. ^b^ Epigenetic effects are not displayed; they may occur through exposures at each step along the causal pathways.

Using the analogy of a river flowing downstream to its destination from its upstream source, an upstream cause is one that acts at the beginning of a long causal chain, setting in motion multiple midstream and/or downstream causes, which then more directly trigger the physiological mechanisms that directly produce PTB. This is illustrated by Figure 1, which, based on the literature, is a greatly simplified illustration of some of the many plausible pathways through which racism, as an upstream causal factor, may result in the racial disparity in PTB by activating midstream and downstream causes. For example, gerrymandering and voter suppression are products of racism that lead to African Americans lacking political representation. Lack of political representation puts African Americans at a disadvantage economically and environmentally because it means that their interests are not being championed and addressed, and resources are not being allocated to their communities (midstream factors). This in turn results in a range of harmful exposures downstream that directly trigger physiological mechanisms resulting in PTB.

Also illustrated by Figure 1, racism could contribute to the racial disparity in PTB—and other important health outcomes—through racial residential segregation. Discrimination in housing is no longer legal, but the legacy of Jim Crow laws endures in pervasive racial segregation. US census data for 2013‐2017 reveal that on a national basis, 52.6% of African Americans would have to move to achieve full integration with Whites;^70^ in some cities, three out of four African Americans would need to move in order to achieve full integration.^71^ Segregation tracks African Americans into neighborhoods that are less healthy in many ways, including exposure to pollution due to environmental injustice,^49^, ^72^ exposure to unhealthy foods,^73^, ^74^ and the stress of living in deteriorating areas with concentrated poverty.^75^ This affects not only poor but also many middle‐class African Americans.^76^, ^77^ Segregation cuts African Americans off from services, homeownership, good schools, decent jobs, and economic opportunities.^78^ Low levels of wealth^79^ or income^80^ and inferior schooling^80^, ^81^ result in poor health across generations.

It is important to understand that all of this can happen even without any individual now consciously intending to discriminate, because the factors involved reflect systemic and structural racism, that is, the racism built into systems and structures, including laws, policies, widely established practices, and entrenched beliefs. These include the policies, practices, and beliefs driving discriminatory treatment of African Americans by banking institutions, which are obstacles to homeownership and the ability to start or expand a business, and therefore to accumulate wealth.^75^ Wealth, which is more likely to reflect economic resources across a person's entire life course, may be even more important to health than income, which measures economic resources during a limited period of time.^79^

Discriminatory structures associated with racial residential segregation also include underresourced public schools. The schools are underresourced because low wealth means low property taxes, an important source of support for schools; education is key to employment opportunities. Racism also constrains the economic opportunities of African Americans through discrimination in hiring and promotions.^82^, ^83^

Lack of socioeconomic resources due to any discriminatory cause could affect PTB risk in many ways. Discriminatory causes could include biased lending practices, employment discrimination, inferior schools, and/or biased policing and sentencing practices; the latter stigmatize people for life, blocking their access to employment and housing opportunities.^84^, ^85^, ^86^ Consequences of limited socioeconomic resources could include inability to purchase a nutritious diet,^73^, ^74^ toxic exposures (because of inability to afford housing in an environmentally safe area),^49^, ^72^ and the constant stress associated with facing ongoing daily challenges like child care, transportation, and feeding and sheltering one's family, without adequate resources. Most studies, however, measure socioeconomic factors very inadequately, often just one (typically current income or educational attainment) or both measures, which is not enough; the racial disparity in PTB has been observed to be reduced by about half after adjusting for more than two socioeconomic variables.^28^

Stress, including stress unrelated to economic hardship, also appears likely to play an important role in the Black‐White disparity in PTB. Neuroscience has revealed that psychosocial stress (“stress”) occurs when life's demands (stressors) strain or exceed a person's adaptive resources, resulting in downstream biopsychosocial responses (the stress response) that can compromise health.^87^, ^88^ Considerable research links stress to PTB through stress‐induced physiological mechanisms,^89^, ^90^, ^91^, ^92^ including inflammation and immune dysregulation.^93^ Stress, furthermore, influences behaviors;^94^, ^95^ many plausible downstream factors have potentially substantial behavioral influences. Although conclusions are not definitive in every case, studies have linked stress, directly or indirectly, to many plausible downstream factors, including diet/nutrition;^96^ infection;^92^ microbiota;^97^, ^98^ obesity;^99^ prepregnancy hypertension;^100^ hypertensive disorders of pregnancy (HDP);^101^ prepregnancy diabetes;^102^ gestational weight gain;^103^ gestational diabetes;^104^ and epigenetic effects.^105^

Prepregnancy hypertension and HDP, such as preeclampsia, are more common among African American women and have been strongly associated with PTB.^106^, ^107^, ^108^, ^109^ The direct contribution of stress to the development of hypertension remains equivocal due to methodological inconsistencies in the research.^100^, ^110^, ^111^, ^112^ Inflammation, however, which can result from chronic stress‐related immune dysregulation,^93^ has repeatedly been linked to hypertension in both pregnant and nonpregnant women.^113^, ^114^, ^115^ Inflammation is thought to be involved in HDP;^116^, ^117^ preeclampsia itself has been considered “an excessive maternal inflammatory response to pregnancy.”^118^ A recent study in the Netherlands found elevated hair cortisol levels (measured from three months preconception to the end of the second trimester) and anxiety scores (at hospital admission) in women with preeclampsia, a strong predictor of PTB.^119^

Although African American women generally report more stress,^120^, ^121^, ^122^ this has not consistently explained PTB disparities.^123^, ^124^, ^125^ This may be due to studies' inadequate measurement of stress and/or an exclusive focus on stress during pregnancy rather than throughout the life course.^60^, ^61^ The physiologic “wear and tear” caused by chronic stress can compromise women's reproductive health long before they become pregnant.^126^ Few studies, however, consider exposure of women across the entire life course, including in childhood, to chronic social stressors such as racism and/or its effects. A systematic review concluded that chronic stress is more likely than acute stress to result in prolonged elevated blood pressure,^112^ supporting the hypothesis that experiences across the life course may be more important than experiences during pregnancy alone. Many researchers have concurred that there is likely an important, biologically plausible role for stress across a woman's life course (but not necessarily during pregnancy) in the PTB disparity.^62^, ^63^, ^64^, ^65^, ^66^, ^127^, ^128^

## Race—or Racism? Unmeasured Social Differences

All too often, and generally unconsciously, when health researchers observe that a variable representing “race” is associated with a health outcome, they infer that the “race” variable reflects biological differences, particularly if they have adjusted for current income and/or educational attainment. The overwhelming scientific consensus today, however, is that “race” is a social, not a biological, construct.^11^, ^12^, ^13^, ^16^ “Race” is presented within quotation marks here as a reminder of its socially constructed nature.^11^, ^12^, ^13^, ^14^, ^15^

All too often researchers who have concluded that “race” represents biological and/or behavioral differences have failed to consider unmeasured social differences such as those discussed earlier in the paper; this may lead to erroneous conclusions about the racial disparity in PTB and other health outcomes. As noted, structural and systemic racism have constrained African Americans’ economic opportunities—resulting in less favorable education, employment, income, wealth, and neighborhood conditions, starting in infancy. Despite this, all too often scientific papers conclude that an observed racial difference in health must reflect underlying biological differences or behavior because the researchers saw a racial difference after they “controlled for socioeconomic status (SES).”^129^ It is not possible to control for SES. It is too multifaceted; it is not just a person's current income or education but both, as well as their accumulated wealth, the quality of their education and the prestige associated with it, their neighborhood socioeconomic conditions (e.g., concentrated poverty/deprivation versus concentrated privilege, environmental exposures), and their parents’ wealth, education, and income, which powerfully shaped their experiences and exposures when they were children. Socioeconomic conditions during childhood may have particularly strong effects.^130^ Socioeconomic status encompasses all of these factors and more throughout a person's life, and no researcher can capture all of it. Because of racism, furthermore, at the same level of education, African Americans have far less income than Whites.^28^ And at the same income, African Americans live in poorer neighborhoods and have a fraction of the accumulated wealth.^131^ All of these differences can affect health in general and PTB in particular. These factors are rarely measured, yet studies often conclude a racial difference is genetic if it persists after “controlling for SES.” The “race” variable often captures unmeasured socioeconomic, psychosocial, and environmental factors.

Racism also could plausibly affect PTB not only through limiting a person's economic opportunities but also through the direct psychological effects of experiencing, anticipating, or being aware of unfair treatment of one's racial group. All of these can be stressful, regardless of one's socioeconomic resources. It is not just dramatic incidents. It is the cumulative effects of daily experiences that may be subtle or ambiguous, the effects of having to be constantly vigilant for a slight or insult—intended or not—and the cumulative effects of countless little assaults on one's self‐esteem. Or it may be the stress experienced by a woman learning that yet another unarmed Black man has been killed by the police and constantly wondering whether her husband or son is next. Chronic stress, even at a low level, is biologically plausible as a contributor to PTB through neuroendocrine and immune mechanisms. Neuroscientists have identified physiologic pathways leading from stress to health damage, including effects on inflammation and immune system functioning known to trigger labor. A large population‐based study found that the Black‐White disparity in PTB was no longer significant after adjusting for whether a woman often worried about being treated unfairly based on her race.^53^ As noted earlier, several studies have observed a wider racial disparity in PTB among more socioeconomically advantaged women than among their less‐advantaged counterparts; possible explanations include greater chronic stress among higher‐income or higher‐education Black women due to paradoxically greater exposure to chronic racism at work, where they are likely to be in the minority.^53^, ^132^, ^133^ Other hypotheses include the psychosocial price African Americans may pay for upward social mobility, including constantly feeling they must try harder than their White peers to overcome stereotypes.^132^, ^133^ Very few health studies have measured people's stressful experiences of racism, which are a biologically plausible and potentially very important difference.

Racism may therefore be an important contributor to the Black‐White difference in PTB and other adverse health outcomes, through many pathways, including those noted here and others. Health‐damaging (or lack of health‐promoting) pathways may operate even when no one currently intends to discriminate, because they are the effects of deeply embedded structural or systemic racism. None of these pathways is usually measured adequately, if at all, in health studies, yet this limitation is rarely acknowledged. It should be noted, however, that many of the issues discussed in this paper, while insufficiently considered in most health research, have been more adequately addressed for some time in the social science and humanities literatures.^14^, ^16^, ^134^, ^135^, ^136^, ^137^, ^138^

While this paper focuses on preterm birth, the key arguments, particularly about plausible causal pathways from racism to health damage and unmeasured differences—are relevant to a wide range of health outcomes, particularly cardiovascular disease and diabetes, where the roles of inflammation and immune dysfunction are believed to be important, as they are in PTB. The implications of this paper therefore extend far beyond PTB. The practical implications apply to all those who read and/or conduct studies that include variables representing “race.” One must be aware that the “race” variable is always picking up the totality of unmeasured experiences that a person of that “race” may have endured, throughout that individual's life course, which could have influenced health. And even the most knowledgeable, skilled, and determined researcher could have measured at best only a tiny fraction of those experiences. When reading, designing, or analyzing results of a study of any health outcome that includes a variable representing “race,” one should always ask: are we looking at the effects of “race”—or of racism?

Addressing structural and systemic racism and their cascading effects is fundamental to improving population health and reducing health inequities, as discussed in greater detail by Brown and Hohlman,^139^ and also by Michener and Ford in this special issue.^140^ In addition, as Ray, Lantz, and Williams argue,^141^ a priority agenda for promoting health equity must include reducing racial discrimination (structural as well as interpersonal) both in and far beyond the health care system.
